# Right Heart Chambers Longitudinal Strain Provides Enhanced Diagnosis and Categorization in Patients With Pulmonary Hypertension

**DOI:** 10.3389/fcvm.2022.841776

**Published:** 2022-03-31

**Authors:** Nilda Espinola-Zavaleta, Neftali Eduardo Antonio-Villa, Enrique C. Guerra, Navin C. Nanda, Lawrence Rudski, Ricardo Alvarez-Santana, Gyssele Camacho-Camacho, Alberto Aranda-Fraustro, Jorge Cossio-Aranda, Karina Zamora, Diego Oregel-Camacho, Javier Ivan Armenta-Moreno, Joaquin Berarducci, Erick Alexanderson-Rosas

**Affiliations:** ^1^Department of Nuclear Cardiology, National Institute of Cardiology Ignacio Chavez, Mexico City, Mexico; ^2^Department of Echocardiography, The American British Cowdray Medical Center, Private Assistance Institution, Mexico City, Mexico; ^3^MD/Ph.D. (PECEM) Program, Facultad de Medicina, Universidad Nacional Autonoma de Mexico, Mexico City, Mexico; ^4^Division of Cardiology, Department of Medicine, University of Alabama, Birmingham, AL, United States; ^5^Jewish General Hospital, McGill University, Montreal, QC, Canada; ^6^Inter-Institutional Program for Strengthening Research and Postgraduate Studies in the Pacific (Dolphin), Mexico City, Mexico; ^7^Department of Pathology, National Institute of Cardiology Ignacio Chavez, Mexico City, Mexico; ^8^Out-Patient Clinic, National Institute of Cardiology Ignacio Chavez, Mexico City, Mexico; ^9^Department of Physiology, School of Medicine, National Autonomous University of Mexico, Mexico City, Mexico

**Keywords:** echocardiography, right ventricular free wall longitudinal strain, right atrial global longitudinal strain, pulmonary hypertension, systolic pulmonary arterial pressure

## Abstract

**Background:**

Increased systolic pulmonary arterial pressure (sPAP) could lead to the mechanical dysfunction and myocardial fibrosis of the right heart chambers. Echocardiographic strain analysis has not been adequately studied in patients with pulmonary hypertension (PH).

**Study design and methods:**

A cross-sectional cohort of patients with suspected PH and echocardiographic strain evaluation was recruited. The cut-off values of peak tricuspid regurgitation velocity (TRV) with the low probability of PH (≤2.8 m/s), intermediate probability (2.9–3.4 m/s, without other echo PH signs), and high probability of PH (2.9–3.4 m/s with other echo PH signs and >3.4 m/s) categories were studied by right ventricular and right atrial (RA) strain analysis in a sample of 236 patients.

**Results:**

The results showed that 58 (56.9%) patients had low, 15 (14.7%) had intermediate, and 29 (28.4%) had a high probability of PH. We observed a negative association between right ventricular free wall strain (RV-FWS) and atrial global strain with sPAP. With the increase in PH severity, RA reservoir, conduit, and contraction (booster) strain values decreased. The identified cut-off values of strain parameters had an adequate ability to detect PH severity categories. In addition, the post-mortem biopsies of right heart chambers from subjects with known severe PH were analyzed to quantify myocardial fibrosis. Our sample of right heart biopsies (*n* = 12) demonstrated an association between increased sPAP before death and right ventricular and RA fibrosis.

**Conclusion:**

Mechanical dysfunction and fibrosis in the right chambers are associated with increased sPAP. Right ventricular and atrial strain could provide enhancement in the diagnosis and categorization of subjects with suspected PH.

## Highlights

- Right heart echocardiographic strain analysis to enhance the diagnosis of pulmonary hypertension (PH) is an area of opportunity.
- We assessed the performance of the deformation parameters of the right heart chambers in characterizing the different severity levels of PH.
- The use of right ventricular and right atrial (RA) strain could provide enhancement in the diagnosis and categorization of subjects with suspected PH.

## Introduction

Echocardiography remains a fundamental clinical imaging tool for the assessment of the right ventricle (RV) in the heart. Conventional echocardiographic parameters used in daily clinical practice that evaluate RV systolic function include tricuspid annular plane systolic excursion (TAPSE), the maximum velocity of the tricuspid lateral annulus during systole or the S wave (S’), and RV fractional area change (RVFAC) (1). All these echocardiographic parameters have well-known limitations (2). The assessment of right ventricular free wall longitudinal strain (RV-FWS) by the two-dimensional echocardiographic speckle tracking analysis has overcome some of these limitations and has emerged as a feasible and reproducible parameter to evaluate RV systolic function (3, 4). RV-FWS has demonstrated a good prognostic value in different clinical scenarios, such as heart failure and congenital heart disease (3). Nonetheless, RV-FWS and right atrial (RA) global strain (RA-GS) have not been fully explored in a broad variety of pathologies (5–8). In patients with pulmonary hypertension (PH), RV-FWS has been shown to be a potential predictor of major cardiovascular events. Moreover, its validation has been assessed with gold-standard methods, such as cardiac MRI (CMR) (9). However, to date, the diagnostic and predictive value of RV-FWS and RA-GS in PH has not been fully explored. The use of the right heart strain parameters in a clinical setting could broaden the stratification and overall, bring relevant information for care providers in patients with PH. Furthermore, the evaluation of the long-standing effect of increased systolic pulmonary arterial pressure (sPAP) and the myocardial fibrosis of the right heart chambers could support the hypothesis that myocardial deformation should be promptly tested in the early stages of patients with PH. Therefore, the main aim of this study is to assess the correlation of RV and RA strain with sPAP parameters. As a secondary objective, we evaluate the ability of strain parameters to predict PH and to categorize its severity compared with other echocardiographic parameters. Furthermore, we extracted post-mortem sample biopsies to measure the degree of myocardial fibrosis in 12 patients classified with severe PH to establish the association of increased sPAP with fibrosis.

## Materials and Methods

### Study Population Cohorts

We designed a cross-sectional study in which we recruited consecutive patients who were evaluated in the echocardiographic division from the Nuclear Cardiology Department (NCD) at the National Institute of Cardiology Ignacio Chavez, Mexico, between the period of June 2018 and December 2019. The patients attended our institution’s outpatient clinic due to dyspnea on exertion, fatigue, and dizziness and were sent for a transthoracic echocardiogram for further evaluation. All patients underwent conventional two-dimensional and Doppler transthoracic echocardiography, along with velocity vector imaging to assess the right heart chamber strain parameters. We excluded subjects with congenital heart diseases, prior myocardial infarction, sarcoidosis, mild or severe valvular disease, or subjects classified with unspecified cardiomyopathies. Patients with low echocardiographic image quality were excluded in the final analysis. We extracted our control group from the same patients who attended our institution’s outpatient clinic. The control group was defined by subjects who had normal pulmonary artery pressure values by echocardiographic measurement of peak tricuspid regurgitation velocity (TRV) (≤2.8 m/s) within our cohort sample. To assess the inter-rater reliability and reproducibility of echocardiographic sPAP parameters, 13 selected subjects from our first cohort were assessed at heart catheterization performed 10 days after the echocardiographic study. Written informed consent was obtained from all participants.

### Echocardiographic Assessment

We performed a complete conventional transthoracic echocardiogram with subjects in left lateral decubitus using a Siemens Acuson SC 2000 (Mountain View, CA, United States) echocardiographic equipment with a phased array transducer. The right ventricular end-diastolic diameter was measured in the apical four-chamber view, below the tricuspid valve. The RV wall thickness was measured by 2D echocardiography in the subcostal four-chamber view. RA volume was obtained using a single-plane method of disks in the apical four-chamber view at ventricular end-systole, and it was indexed by body surface area (BSA). The measurements of RVFAC, TAPSE, tricuspid S-wave velocity, Tei index, E, A-wave velocities (rapid filling and atrial contribution, respectively), E/A ratio, and tricuspid E/e′ ratio were obtained according to the guidelines of the American Society of Echocardiography and the European Association of Echocardiography (5, 10, 11).

### Pulmonary Arterial Pressure Assessment

The sPAP was calculated by peak TRV with continuous-wave Doppler in the apical four-chamber view, using the simplified Bernoulli equation: 4 × (maximal TRV)^2^ + right atrial pressure. RA pressure was estimated in the subcostal view according to inferior vena cava (IVC) size and collapsibility following a normal sniff: An IVC diameter <2.1 cm that collapsed >50% with a sniff suggested normal RA pressure of 3 mm Hg (range, 0–5 mmHg), whereas an IVC diameter >2.1 cm that collapsed <50% with a sniff suggested a high RA pressure of 15 mmHg (range, 10–20 mmHg). In scenarios in which the IVC diameter and collapse did not fit this paradigm, an intermediate value of 8 mmHg (range, 5–10 mmHg) might be used, or, preferably, other indices of RA pressure could be integrated to downgrade or upgrade to the standard or high values of RA pressure (5, 11). The echocardiographic probability of PH was classified as (1) low: peak TRV ≤ 2.8 m/s, (2) intermediate: peak TRV 2.9–3.4 m/s, without other echo PH signs, and (3) high: peak TRV 2.9–3.4 m/s with other echo PH signs and >3.4 m/s, based on the 2015 European Society of Cardiology (ESC)/European Respiratory Society (ERS) Guidelines for the diagnosis and treatment of PH (12).

### Echocardiographic Strain Assessment

Strain assessment was performed offline using velocity vector imaging (Siemens Acuson SC 2000, version 5). All images analyzed were obtained at 50–80 frames/s at end expiration. The region of interest was traced with a point-and-click approach on the endocardium of the RV free wall at end-diastole in the RV-focused apical four-chamber view. A broader region of interest was subsequently generated and manually adjusted if necessary. The program automatically divided the RV free wall into three segments and performed the analysis of the deformation frame by frame. This process allowed an automated confirmation of the contour and generated deformation values. The peak strain values from the three free wall segments were averaged, and the mean value was taken as the right ventricular free wall strain (RV-FWS) (5, 7, 11–13).

**FIGURE 1:**
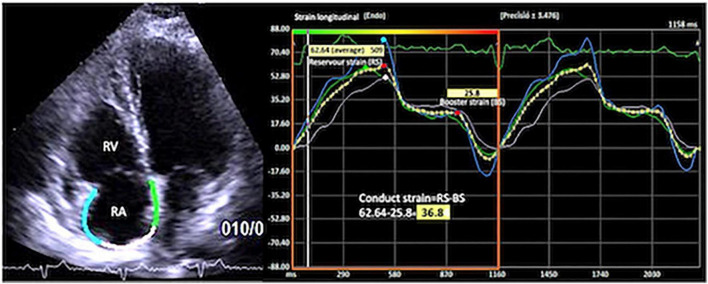
Apical four-chamber view showing the right atrial global longitudinal strain (RA-GS) with the measurement of reservoir, conduit, and contractile strain phases.

For the right atrium (RA), the endocardial border was traced in the apical four-chamber view, excluding the appendage and the Eustachian valve from the RA cavity. RA longitudinal strain curves were generated throughout the cardiac cycle with R-R gating. The accuracy of the automated border tracking was verified and manually adjusted if needed. Tracking was repeated three times, and averages were used for analysis as reported in guidelines (5, 8, 11). The peak RA reservoir strain in ventricular systole, conduit strain in early diastole, and peak contractile phase strain during atrial systole/late diastole were measured and expressed as percentage. The RA total reservoir phase and RA contractile phase were assessed by measuring the corresponding peak strains. The conduit strain was calculated as the difference between RA total reservoir strain and RA contractile strain (Figure 1).

To assess the intra- and inter-observer reproducibility of RA reservoir, conduit, and contractile strain, 13 randomly sampled analyses were repeated two times by the same observer and by a second observer without the knowledge of previous findings, respectively.

#### Postmortem Right Heart Sample Cohort

The second cohort of 12 biopsies of the right heart chambers from the postmortem heart samples of patients diagnosed with severe PH were included. This cohort was created to evaluate the hazardous effect of increased sPAP on the development of fibrosis in the right heart chambers.

The heart was photographed, the macroscopic characteristics were taken, and sections were made for histological study. Samples were taken from the RA and the right ventricular free walls. We took photographs of the longitudinal section from the atrium’s anterior wall, from the origin of the appendage to the tricuspid valve, and a transverse section of the ventricle in the middle portion of the free wall, covering the entire thickness of the wall.

**FIGURE 2:**
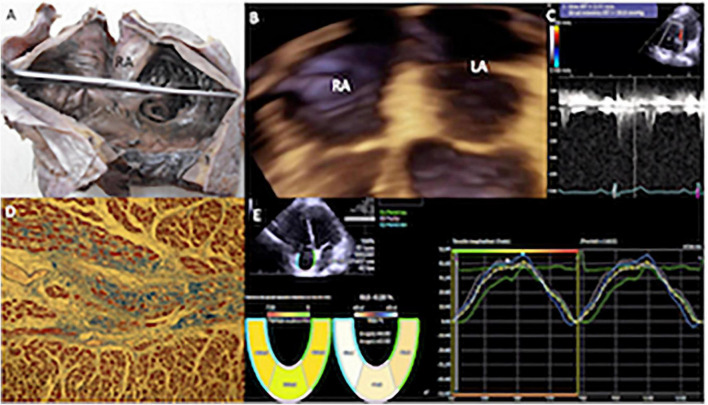
**(A)** Specimen showing the internal appearance of the dilated right atrium, the pectinate portion that continues with the appendage is observed to the right of the observer, and the anterior leaflet of the tricuspid valve is observed below. **(B)** Transthoracic 3D echocardiogram in four-chamber view with mild enlargement of the right atrium. **(C)** Mild pulmonary hypertension (PH) systolic pulmonary arterial pressure (sPAP) of 44 mmHg. **(D)** Histological study (stained with Masson, 10×) of the atrial myocardium of a patient, with mild PH. The muscle fascicles were mostly cut transversely or obliquely. Two muscle fascicles are replaced by fibrous connective tissue rich in collagen fibers that stain blue, and contrast with the red in which the myocardium is stained. One fascicle is partially replaced by collagen and another almost entirely. The degree of fibrosis in the observed fields was calculated at 25%, since in this image it could reach a little more than a quarter. **(E)** Echocardiography with velocity vector imaging of a patient with a low probability of PH, who had a normal global longitudinal strain of the right atrium (43.5%). RA, right atrium; RV, right ventricle.

**FIGURE 3:**
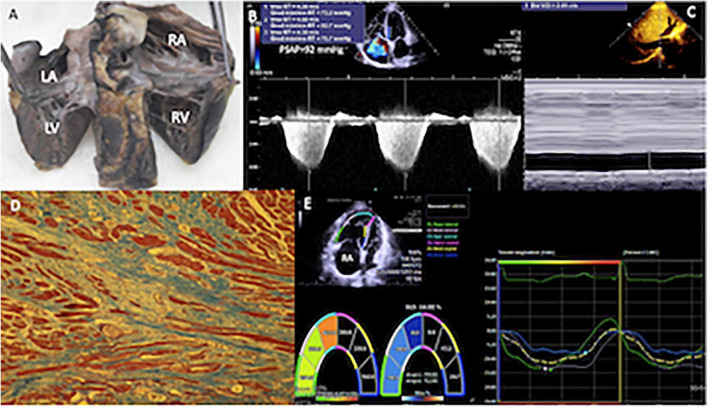
**(A)** Specimen of the heart of a patient with sPAP of 90 mmHg. Heart cut longitudinally from the atria to the ventricles (entry routes through the posteroinferior aspect) that allow to appreciate the dilation of the right cavities. **(B)** Two-dimensional echocardiogram with Doppler in the apical four-chamber view, with severe tricuspid regurgitation, by means of which an average gradient of 77 mmHg was calculated. **(C)** In the subcostal plane, the dilatation of the inferior vena cava (29.1 mm) and collapse of <50% were detected, calculating a right atrial pressure of 15 mmHg. **(D)** The histological study of right ventricular myocardium, most of the muscle fibers are in longitudinal section in red and the fibrous tissue rich in collagen fibers is observed in blue, it extensively replaces the muscle fascicles, and, in this image, it reaches 75% or three quarters. The muscle fibers embedded in the fibrous connective tissue appear elongated and thinned. Masson 10×. **(E)** The global longitudinal strain of the right ventricle, using velocity vector imaging was −14%, in a patient with a high probability of PH (sPAP = 92 mmHg). RA, right atrium; RV, right ventricle; LA, left atrium; LV, left ventricle.

The samples were processed with the histological technique of “paraffin inclusion.” They were stained with the Masson technique to quantify the percentage of RA and right ventricular fibrous tissue, dividing the field of observation of the microscope into quarters. Two independent observers gave the percentage values, and a consensus value was obtained when there were differences. Microscopic photographs were taken of the most representative areas (Figures 2, 3).

The study was carried out following the Declaration of Helsinki and was approved by the Ethics and Research Committee of the National Institute of Cardiology Ignacio Chavez. Reference number: PT-17-087.

### Statistical Analysis

The frequency distribution of categorical variables is reported as frequencies and percentages. Data are presented as mean (SD) or median [interquartile range (IQR)] where appropriate. To compare the differences of echocardiographic parameters among PH categories, we performed a one-way ANOVA or Kruskal–Wallis test wherever it met assumptions of parametric tests or not, and Dunn’s *post hoc* test was also assessed to evaluate the differences among groups.

#### Correlation of Strain Parameters With a Probability of PH

We performed a natural logarithmic transformation in variables with the non-parametric distribution. Afterward, we assessed the correlation of both RV-FWS and RA-GS with sPAP using Pearson’s correlation analysis to obtain the correlation coefficient with our transformed variables. To evaluate the prediction capacity of RV-FWS with sPAP, we performed polynomic adjusted linear regression analysis to assess the association between both parameters. The *R*^2^ was reported to express the variability explained by both variables. As a second step, we adjusted these models for age, sex, and BSA as these variables could modify the relationship between RV-FWS and sPAP. As a secondary analysis, we evaluated the association of sPAP with the right heart chamber fibrosis of our second cohort sample, using the methods previously described.

#### Diagnostic Performance of Right Heart Chambers Strain Parameters

We sought to evaluate the diagnostic performance of RV-FWS and RA-GS to predict categories of PH severity. Receiver operating characteristic (ROC) analysis curves were generated and area under the curve (AUC) derived for RV and RA strain and compared with the Fraction of Shortening, TAPSE, TEI, which are commonly used echocardiographic parameters to evaluate right heart ventricular function. Furthermore, we sought to identify the optimal cut-off value of the strain parameters using the “Youden method” from the R package “*Optimal cut points*” and evaluate the diagnostic test capacity, AUC, sensitivity, specificity, and positive and negative predictive values (VPP and VPN, respectively) to predict PH categories (14). Finally, we performed logistic regression models to assess the likelihood to have each PH category with their respective identified cut-off value. The goodness of fit of the logistic regression model was assessed using the Hosmer–Lemeshow test. All statistical analyses were performed using the R software (version 3.5.1) (15). A value of *p* < 0.05 was considered as our statistically significant threshold.

## Results

### Study Population

We evaluated 314 patients in our study period, of which 236 had completed clinical and echocardiographic evaluation parameters for our main analyses (Supplementary Figure 1). The demographic and echocardiographic assessments of our first cohort sample are presented in Table 1. Briefly, our population had a male predominance (52.9%), with a mean age of 55 (±15) years. Arterial hypertension was recorded in 96 (40.7%) patients, followed by obesity in 90 (38.1%), diabetes mellitus in 80 (33.9%), and 42 (17.8%) with dyslipidemia and previous myocardial infarction. The echocardiographic evaluation showed a median peak RV-longitudinal FWS of −26.9% (IQR: −31.2 to −21.2%) and a peak RA-GLS of 42.2% (IQR: 30.6–55.0%). Median sPAP was 33 (IQR: 28–41) mmHg. In our studied sample, 134 (56.8%) had normal sPAP values from peak TR velocity, which represented our control group; 102 (43.2%) were classified with the probability of PH. Of these patients, 58 (56.9%) had low, 15 (14.7%) intermediate and 29 (28.4%) had a high probability of PH. We observed an intraobserver and interobserver variation of 9 and 5%, respectively. Finally, in the 13 patients submitted to cardiac catheterization, we observed an acceptable inter-rater reliability coefficient (IRC: 56.8%) with echocardiographic sPAP parameters and an overall variance with the mean between both parameters ≤30%. The correlation of the echocardiogram with the cardiac catheterization in the determination of pulmonary arterial systolic pressure was of *r* = 0.777 (Supplementary Figure 2).

**TABLE 1:** Demographic and echocardiography assessment of study population.

Parameter	*n* = 236
Male (%)	124 (52.9%)
Age (years)	54.6 (±15.6)
Height (cm)	1.62 (±0.1)
Weight (kg)	71.8 (±15.4)
BSA (cm)	1.75 (1.62–1.90)
Obesity (%)	90 (38.1%)
Arterial hypertension (%)	96 (40.7%)
Diabetes (%)	80 (33.9%)
Dyslipidemia (%)	42 (17.8%)
Previous myocardial infarction (%)	42 (17.8)
Tricuspid regurgitation (%)	158 (66.9%)
Mild-TR (%)	138 (58.5%)
Moderate-TR (%)	14 (5.9%)
Severe-TR (%)	6 (2.5%)
**Right ventricle**	
RVd (mm)	36 (33–40.2)
RVFAC (%)	40.4 (35–48)
TAPSE (mm)	20 (17.4–22)
RV-synchrony (ms), *n* = 141	22 (3.5–44)
TEI index	0.53. (±0.17)
E Wave (cm/s), *n* = 141	9.0 (7–12)
A Wave (cm/s), *n* = 141	13 (9.25–16)
S Wave (cm/s), *n* = 141	11 (9.8–12.4)
E/A, n = 141	0.76 (0.62–0.94)
**Right atrium**	
Volume (ml/m^2^)	31 (21–44)
Area (cm^2^)	14 (11.6–17)
Reservoir phase (%)	41.7 (30.3–55)
Conduit phase (%)	22 (13.4–30)
Contractile phase (%)	18.8 (13.3–26.9)
sPAP (mmHg)	33 (28–41)
**PH categories**	
No-PH (%)	131 (55.5)
With-PH	102 (43.2)
Mild (%)	58 (56.9)
Moderate (%)	15 (14.7)
Severe (%)	29 (28.4)
**Ventricular and atrial strain**	
RV-FWS (%)	−26.86 (−21.2 to −31.22)
RA-GS (%)	42.2 (30.6–55)

*BSA, body surface area; RVd, right ventricle diameter; FAC, fractional area change; TAPSE, tricuspid annular plane systolic excursion; RVS, right ventricular Synchrony; TR: tricuspid regurgitation.*

### Association of Strain Parameters With Probability of PH

**FIGURE 4:**
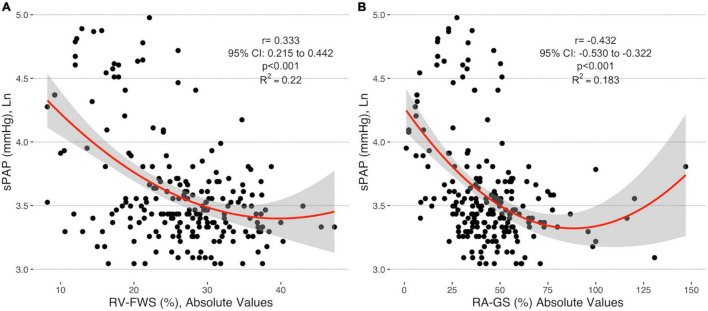
Correlation of the absolute values of RV-FWS **(A)** and RA-GS **(B)** with logarithmical sPAP. sPAP, systolic pulmonary arterial pressure; RV-FWS, right ventricular free wall strain; RA-GS, right atrial global longitudinal strain.

**FIGURE 5:**
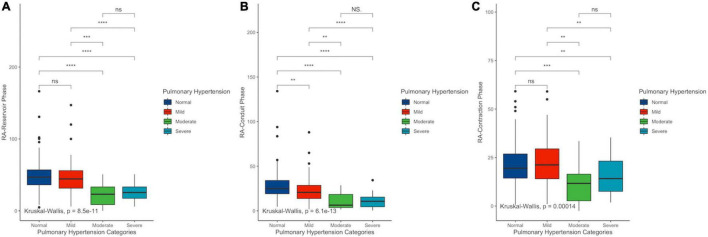
Differences among right atrial reservoir **(A)**, conduit **(B)**, and contractile **(C)** phases in PH categories. sPAP, systolic pulmonary arterial pressure; RV-FWS, right ventricular free wall strain; RA-GS, right atrial global longitudinal strain. **p < 0.01, ***p < 0.001, and ****p < 0.0001.

Right ventricular free wall strain absolute values were negatively correlated with sPAP (*r* = −0.333, 95% CI −0.215 to 0.442) as well as with RA-GLS (*r* = −0.432, 95% CI −0.530 to −0.322). RV-FWS and RA-GLS explained 22.2 and 18.3% of the variability of sPAP, respectively. These trends were sustained after adjusting for age, sex, and BSA. Interestingly, we observed that both parameters had a quadratic fit adjustment (Figure 4). Regarding the right atrial chamber assessment, the reservoir, conduit, and contractile phases had decreased parameters with advanced PH categories suggesting a functional and structural decline of the RA function. However, when comparing specifically between the intermediate and high probability of PH groups, these changes were not statistically significant (Figure 5). This might be related to the small number of subjects classified with intermediate PH.

### Association of Probability of PH With Right Chamber Fibrosis

The relationship between increased sPAP values and myocardial fibrosis in right chambers was evaluated in our second cohort of postmortem samples biopsies. Clinical and echocardiographic characteristics are presented in Supplementary Table 1. sPAP had a positive correlation with right ventricular fibrosis (*r* = 0.671, 95% CI: 0.118–0.906; *p* = 0.024), but not with atrial fibrosis (*r* = 0.416, 95% CI: −0.246 to 0.81; *p* = 0.203), which explains the 18.3 and 8.1% of the variability of right ventricular and atrial fibrosis, respectively (Supplementary Figure 3).

### Diagnostic Value of Strain Parameters in the Evaluation of Probability of PH

**FIGURE 6:**
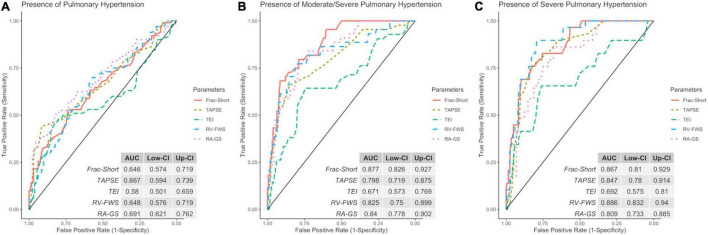
Receiver operating characteristic (ROC) with the area under the curve (AUC) of echocardiographic parameters to identify the discrimination capacity for low **(A)**, intermediate/high **(B)**, and high **(C)** probability of PH. ROC, receiver operator characteristic; Frac-Short, fractional shortening; TAPSE, tricuspid annular plane systolic excursion; TEI, TEI index; RV-FWS, right ventricular free wall strain; RA-GS, right atrial global longitudinal strain.

Finally, as a secondary analysis, we evaluated the ability of strain parameters to predict the presence of PH and to categorize the severity in patients with the probability of PH. Compared with other echocardiographic parameters (RVFAC, TAPSE, and TEI), both RV-FWS and RA-GS showed an adequate AUC to identify the presence of PH and their respective severity categories. RA-GS outperformed other echocardiographic parameters to detect those patients with any degree of PH (AUC: 0.691, 95% CI: 0.621–0.762), while RV-FWS outperformed in those with the high probability of pulmonary hypertension (AUC: 0.886, 95% CI: 0.832–0.940) (Figure 6). RV-FWS of −27.30, −22.60, and −22.10% had an optimal AUC and predictive test performance to predict the presence of PH, and to predict the intermediate-to-high probability of PH, and high PH, respectively. Furthermore, our identified cut-off values for RA-GS were 26.30, 34.36, and 37.20% to detect the previously mentioned categories (Table 2). Using the previously identified cut-off values in our multivariate logistic regression models, we found a significantly increased likelihood for pulmonary hypertension categories, which were maintained after adjustment for covariates (Table 3).

**TABLE 2:** Cut-off values and predictive tests using RV-FWS for pulmonary, intermediate/high, and high probability of pulmonary hypertension (PH).

Outcome	Parameter	Cut-Off	SE	SPE	PPV	NPV	LR+	LR−	AUC
Pulmonary Hypertension	RV-FWS	−27.30	0.700 (0.600–0.787)	0.569 (0.479–0.655)	0.555 (0.465–0.665)	0.711 (0.613–0.780)	1.62 (1.28–2.05)	0.527 (0.37–0.73)	0.648 (0.57–0.72)
Intermediate-High PH		−22.60	0.773 (0.621–0.88)	0.801 (0.736–0.855)	0.479 (0.389–0.676)	0.937 (0.878–0.956)	3.88 (2.79–5.40)	0.284 (0.16–0.49)	0.825 (0.75, 0.9)
High PH		−22.10	0.896 (0.726–0.978)	0.791 (0.728–0.845)	0.382 (0.304–0.761)	0.981 (0.942–0.987)	4.29 (3.19–5.76)	0.131 (0.044–0.38)	0.886 (0.832, 0.94)
Pulmonary Hypertension	RA-FWS	26.30	0.809 (0.731–0.873)	0.515 (0.413–0.615)	0.683 (0.589–0.777)	0.675 (0.572–0.758)	1.67 (1.342–2.072)	0.371 (0.248–0.553)	0.691 (0.621, 0.762)
Intermediate-High PH		34.36	0.793 (0.728–0.848)	0.773 (0.621–0.885)	0.937 (0.878–0.956)	0.466 (0.378–0.664)	3.487 (2.012–6.042)	0.268 (0.194–0.370)	0.84 (0.778, 0.902)
High PH		37.20	0.679 (0.611–0.743)	0.862 (0.683–0.961)	0.972 (0.922–0.979)	0.277 (0.221–0.603)	4.928 (1.97–12.30)	0.371 (0.289–0.476)	0.809 (0.733, 0.885)

*RV-FWS, right-ventricular free-wall strain; AUC, area under the curve; SE, sensitivity; SPE, specificity; PLR, positive likelihood ratio; NLR, negative likelihood ratio; PPV, positive predictive value; NPV, negative predictive value.*

**TABLE 3:** A logistic regression model to predict intermediate/high PH using identified cut-off values for each outcome adjusted for sex, age, and BSA.

Model	Parameter	B	SE	Wald	OR (95% CI)	*P* value
Pulmonary hypertension	RV-FWS	1.402	0.311	4.497	4.06	<0.001
	> −27.30				(2.23–7.63)	
	RA-GS	2.701	0.474	5.699	14.90	<0.001
	< 26.30				(6.22–40.66)	
Intermediate-high probability of pulmonary hypertension	RV-FWS	2.687	0.441	6.088	14.69	<0.001
	> −22.60				(6.43–36.74)	
	RA-GS	2.830	0.457	6.18	16.95	<0.001
	< 34.36				(7.23–44.12)	
High probability of pulmonary hypertension	RV-FWS	3.682	0.683	5.391	39.73	<0.001
	> −22.10				(11.96–187.05)	
	RA-GS	2.778	0.599	4.635	16.10	<0.001
	< 37.20				(5.50–60.48)	

*BSA, body surface area; RV, right ventricle; RA, right atrium.*

## Discussion

In this study, we show the association of both right ventricular free wall and global right atrial strain with increased systolic pulmonary artery pressure. Moreover, we demonstrate that pulmonary arterial hypertension could be associated with the myocardial fibrosis of the right heart obtained with histopathological methods. These findings suggest that the association of increased sPAP values with overall ventricular deformation and fibrosis. Finally, we demonstrate that strain parameters contribute to the detection of PH and the assessment of PH severity in patients with a suspected probability of PH.

The relationship between ventricular deformation and sPAP has been previously reported (16–18). A chronic increase in afterload, manifested by an increased pulmonary artery pressure, can cause a decrease in the elastance of myocardial fibers in patients with severe PH (17, 19). This will ultimately cause irreversible myocardial damage with the eventual development of ventricular fibrosis (20). Our physiopathological hypothesis suggests that ventricular strain and atrial strain are modeled as a quadratic function and these patients have an initial period of compensation by increasing contractility, possibly *via* the Frank-Starling mechanism that progressively decreases as the disease advances. This may be more pronounced in the RV, as the chamber directly faces the increased afterload, before impairing the right atrium. The atrial function is altered as observed in the evaluation of the various atrial phases. Finally, the degree of fibrosis analyzed in the pathological specimens of a subset of patients with PH was associated with a prolonged decrease in ventricular function.

With the demonstration of fibrosis, usually an irreversible change, early detection, and the stratification of PH is critical. Our data demonstrate the clinical utility of RV-FWS and RA-GS as echocardiographic parameters that aid in this task. Strain parameters have been previously used to predict outcomes in congestive heart failure and myocardial infarction with similar results (21, 22), as well as in PH (23–25). The echocardiographic estimation of sPAP and accordingly the development of the probability of PH is usually predicated on the presence of a complete tricuspid regurgitation envelope by continuous-wave Doppler. Often these envelopes are incomplete and the accuracy of the sPAP estimation is markedly reduced. One usually relies on the secondary signs of PH including RV dilatation, dysfunction by TAPSE or S’, or D-shaped septum configuration in systole. Many of these findings are only present in advanced PH. RV strain measurement may permit for earlier detection of dysfunction, as it does in chemotherapy-induced LV cardiomyopathy (26) or in the RV in patients with scleroderma (27). In addition, in the absence of a complete envelope or in the other situations similar to significant regurgitation where stratification into severity categories may not be accurate, strain measurements may similarly assist in this task. In our work, we identified strain cut-off values that demonstrate the differences in PH severity categorization. Overall, RV-FWS offers to be a highly sensitive echocardiographic parameter while RA-GS offers a sufficient specific parameter to detect all the categories of PH. If our cut-off values are validated, they could be used in a clinical setting to aid detection and the categorization of PH. Hence, our results could help the clinicians to further select candidates to be eligible for cardiac catheterization procedures in limited-resource settings.

### Strengths and Limitations

Our study has some limitations. Our patients were recruited at a referral hospital, which may represent a population bias in terms of disease prevalence and severity. Despite this, our population had a significant cohort without PH and had various degrees of pulmonary hypertension. We did not recruit an external cohort to define our control group. Instead, the control group was defined by subjects who had normal pulmonary arterial pressure within our same cohort. Moreover, there was, however, only a small number of patients with moderate PH, potentially affecting our ability to see significantly different measurements between the moderate and severe PH categories. Another potential limitation was the restricted number of patients submitted to cardiac catheterization (*n* = 13), given by the invasive and selective criteria to perform this procedure in all studied population. Furthermore, given the cross-sectional design of our study, we did not assess the etiology of our cases. Nevertheless, according to the evidence published by the “Mexican Registry of Pulmonary Hypertension (REMEHIP),” approximately 43% of all patients with PH in our country had idiopathic PH (28). Additionally, we included a small cohort of patients with PH and with biopsies evaluated postmortem to determine the presence and percentage of fibrosis associated with an increase in sPAP. We did not have strain values in these patients; therefore, the correlation of right heart strain and myocardial fibrosis needs to be further investigated. Accordingly, while fibrosis has been associated with reduced echocardiographic derived strain, we consider the relationship between pathology-derived fibrosis and reduced strain as a measurement of decreased RV function and possibly of fibrosis as exploratory. Finally, the assessment at follow-up to evaluate possible adverse outcomes is left as an area of opportunity for further research.

## Conclusion

Increased pulmonary artery pressure is associated with the dysfunction of the right atrium and RV as shown by decreased RV and RA peak global longitudinal strain. We believe that this chronic dysfunction may be related to an eventual risk for fibrosis. The use of echocardiographic derived strain parameters in clinical practice could be a potential tool for detecting the presence and evaluating the probability of PH as estimated by sPAP. If validated, proposed cut-off values may improve the clinical staging of PH by including a non-invasive marker of dysfunction or fibrosis.

## Data Availability Statement

The original contributions presented in the study are included in the article/Supplementary Material, further inquiries can be directed to the corresponding author.

## Ethics Statement

The studies involving human participants were reviewed and approved by Ethics and Research Committee of the National Institute of Cardiology Ignacio Chavez. Reference number: PT-17-087. The patients/participants provided their written informed consent to participate in this study.

## Author Contributions

NE-Z, NA-V, and EA-R contributed to research idea and study design. RA-S, GC-C, AA-F, and DO-C contributed to data acquisition. NE-Z, NA-V, and EG contributed to data analysis/interpretation. NA-V and EG contributed to statistical analysis. NE-Z, NA-V, LR, EA-R, and NN contributed to manuscript drafting. NE-Z, NN, and LR contributed to supervision or mentorship. All authors contributed to important intellectual content during manuscript drafting or revision and accepts accountability for the overall work by ensuring that questions pertaining to the accuracy or integrity of any portion of the work are appropriately investigated and resolved.

## Conflict of Interest

The authors declare that the research was conducted in the absence of any commercial or financial relationships that could be construed as a potential conflict of interest.

## Publisher’s Note

All claims expressed in this article are solely those of the authors and do not necessarily represent those of their affiliated organizations, or those of the publisher, the editors and the reviewers. Any product that may be evaluated in this article, or claim that may be made by its manufacturer, is not guaranteed or endorsed by the publisher.

## Supplementary Material

Supplementary Figure 1 Algorithm for patients’ selection.

Supplementary Figure 2 Correlation of RV-Fibrosis **(A)** and RA-Fibrosis **(B)** with sPAP. sPAP, systolic pulmonary arterial pressure; RV-FWS, right ventricular free wall strain; RA-GS, right atrial global longitudinal strain.

Supplementary Figure 3 Correlation of sPAP using echocardiographic and right heart catheterization **(A)** and Bland–Altman plot with observed differences between both the methods of measured sPAP **(B)**. sPAP, systolic pulmonary arterial pressure.

## Abbreviations

NYHA: New York Heart Association
RVFAC: right ventricular fractional area change
RV: right ventricle
RV-FWS: right ventricular free wall strain
RA-GS: right atrial global strain
TAPSE: tricuspid annular plane systolic excursion
S’: velocity of the tricuspid S wave
CMR: cardiac magnetic resonance imaging
sPAP: systolic pulmonary artery pressure
LV: left-ventricle
AUC: area under the curve
ROC: receiver operating characteristic
PPV: positive predictive values
NPV: negative predictive values.
