# Supplementary figures and images for: Right Heart Chambers Longitudinal Strain Provides Enhanced Diagnosis and Categorization in Patients With Pulmonary Hypertension
Source: Front Cardiovasc Med. 2022 Mar 31;9:841776. doi: 10.3389/fcvm.2022.841776 (PMC9008240; doi:10.3389/fcvm.2022.841776)

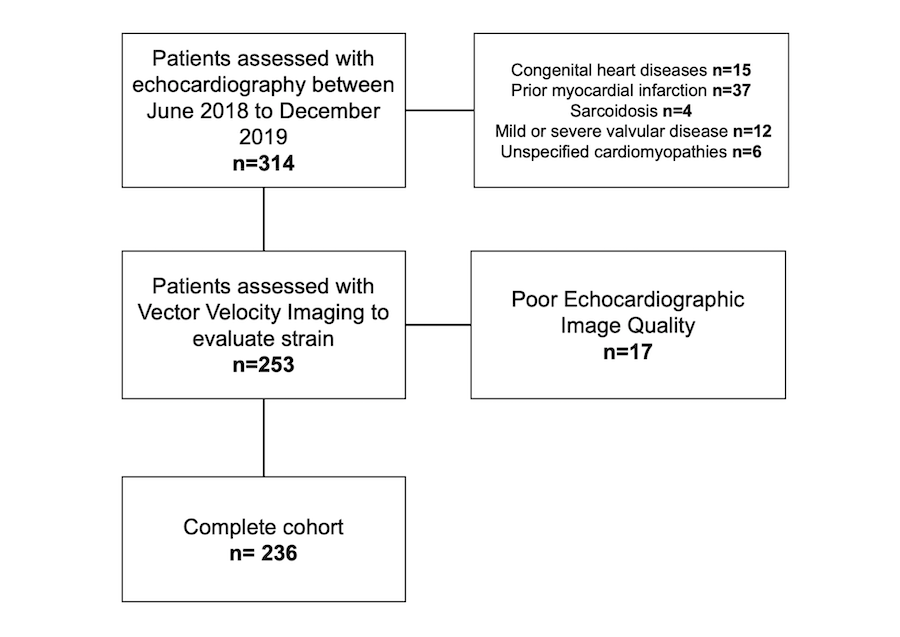

Supplement: Supplementary Figure 1 — Algorithm for patients’ selection. [file Image_1.TIFF]

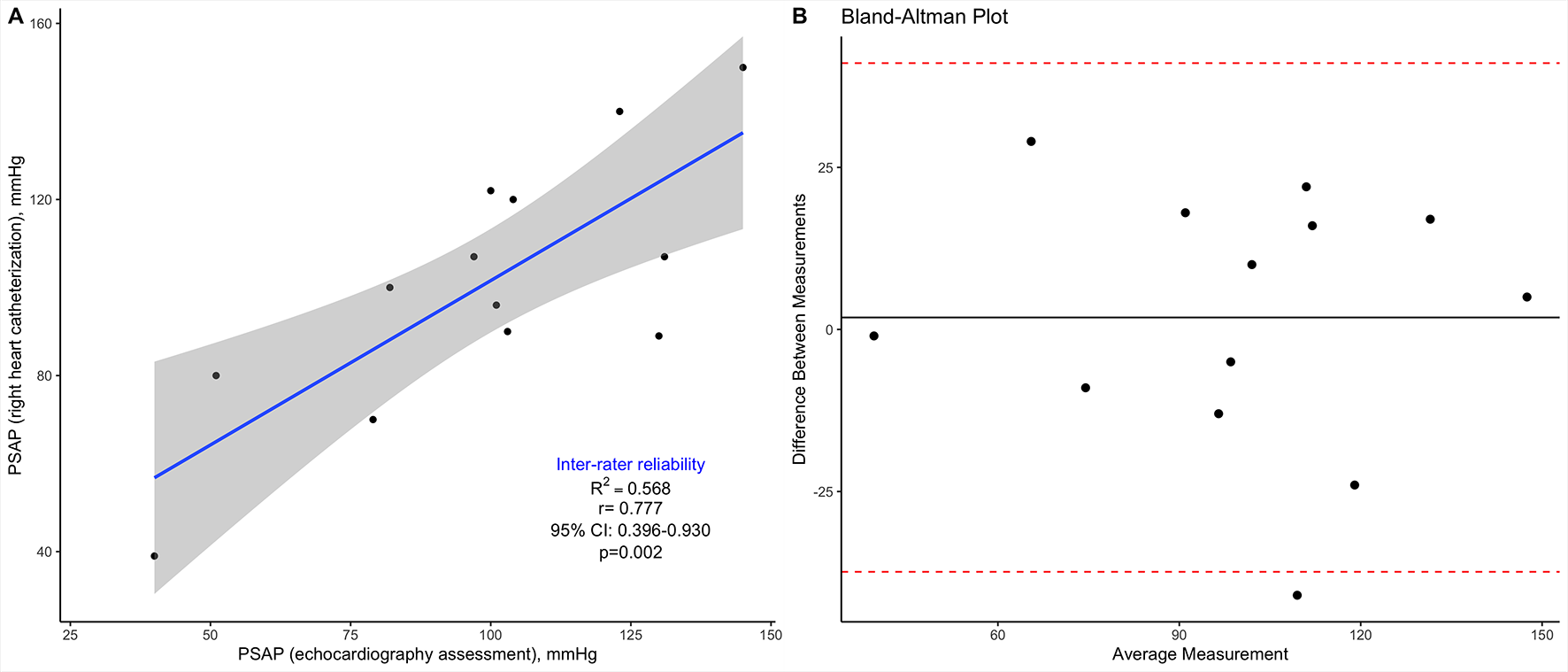

Supplement: Supplementary Figure 2 — Correlation of RV-Fibrosis (A) and RA-Fibrosis (B) with sPAP. sPAP, systolic pulmonary arterial pressure; RV-FWS, right ventricular free wall strain; RA-GS, right atrial global longitudinal strain. [file Image_2.TIFF]

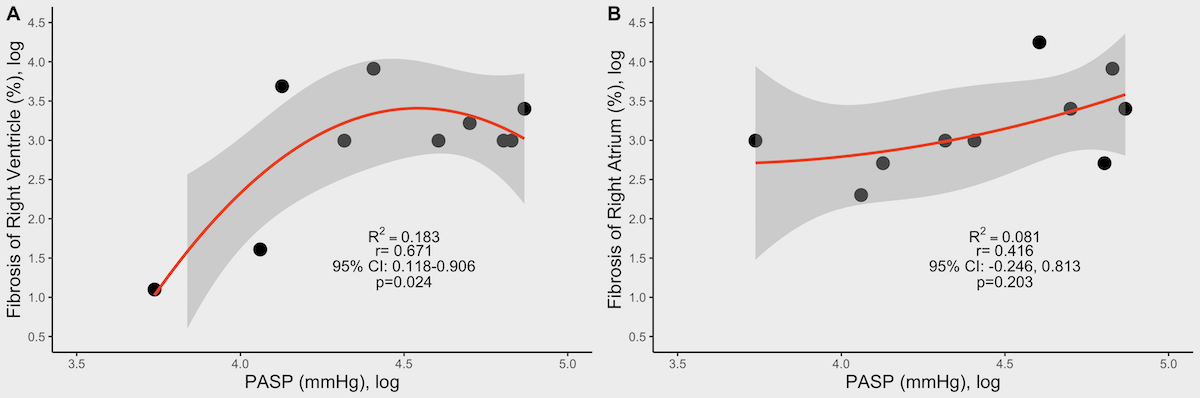

Supplement: Supplementary Figure 3 — Correlation of sPAP using echocardiographic and right heart catheterization (A) and Bland–Altman plot with observed differences between both the methods of measured sPAP (B). sPAP, systolic pulmonary arterial pressure. [file Image_3.TIFF]
