# Supplementary material for: Right Heart Chambers Longitudinal Strain Provides Enhanced Diagnosis and Categorization in Patients With Pulmonary Hypertension
Source: Front Cardiovasc Med. 2022 Mar 31;9:841776. doi: 10.3389/fcvm.2022.841776 (PMC9008240; doi:10.3389/fcvm.2022.841776)
Supplement: Supplementary file 4 [file Table_1.DOCX]

**Supplementary Table 1:** Clinical characteristics of our heart biopsies cohort included to study with severe pulmonary hypertension to study the effect of sPAP and myocardial fibrosis.

| Parameter | n=12 |
| --- | --- |
| Female (%) | 9 (75) |
| Systemic Arterial HTN (%) | 3 (25) |
| Obesity (%) | 1 (8.3) |
| Smoking (%) | 3 (25) |
| Diabetes (%) | 1 (8.3) |
| Dyslipidemia (%) | 1 (8.3) |
| COPD (%) | 0 (0) |
| Chronic Angina (%) | 0 (0) |
| Previous MI (%) | 1 (8.3) |
| Previous Stroke (%) | 0 (0) |
| Valvular Heart Disease (%) | 2 (16.7) |
| Bundle Branch Block (%) | 2 (16.7) |
| Heart Failure (%) | 3 (25) |
| Atrial Fibrillation (%) | 0 (0) |
| Aneurysm (%) | 1 (8.3) |
| Trivascular Disease (%) | 0 (0) |
| Myocarditis (%) | 0 (0) |
| Atrial Septal Defect (%) | 1 (8.3) |
| Ventricular Septal Defect (%) | 0 (0) |
